# Open dorsomedial fracture-dislocation of the medial cuneiform with concomitant tibialis anterior tendon rupture: a case report and review of the literature

**DOI:** 10.1093/jscr/rjag492

**Published:** 2026-06-22

**Authors:** James Muscat, Danil Chernov, Nicholas Frappa, Morgan Dillon, Matthew G Alben, Joshua Slowinski, Christopher A Ritter, Jennifer Gurske-DePerio

**Affiliations:** University at Buffalo Department of Orthopaedics and Sports Medicine, 462 Grider Street, Buffalo, NY 14215, United States; New York Institute of Technology College of Osteopathic Medicine, 101 Northern Blvd, Glen Head, NY 11545, United States; University at Buffalo Department of Orthopaedics and Sports Medicine, 462 Grider Street, Buffalo, NY 14215, United States; Jacobs School of Medicine and Biomedical Sciences, 955 Main Street, Buffalo, NY 14203, United States; University at Buffalo Department of Orthopaedics and Sports Medicine, 462 Grider Street, Buffalo, NY 14215, United States; Jacobs School of Medicine and Biomedical Sciences, 955 Main Street, Buffalo, NY 14203, United States; University at Buffalo Department of Orthopaedics and Sports Medicine, 462 Grider Street, Buffalo, NY 14215, United States; Jacobs School of Medicine and Biomedical Sciences, 955 Main Street, Buffalo, NY 14203, United States; University at Buffalo Department of Orthopaedics and Sports Medicine, 462 Grider Street, Buffalo, NY 14215, United States; Jacobs School of Medicine and Biomedical Sciences, 955 Main Street, Buffalo, NY 14203, United States; University at Buffalo Department of Orthopaedics and Sports Medicine, 462 Grider Street, Buffalo, NY 14215, United States; Jacobs School of Medicine and Biomedical Sciences, 955 Main Street, Buffalo, NY 14203, United States; University at Buffalo Department of Orthopaedics and Sports Medicine, 462 Grider Street, Buffalo, NY 14215, United States; Jacobs School of Medicine and Biomedical Sciences, 955 Main Street, Buffalo, NY 14203, United States; University at Buffalo Department of Orthopaedics and Sports Medicine, 462 Grider Street, Buffalo, NY 14215, United States; Jacobs School of Medicine and Biomedical Sciences, 955 Main Street, Buffalo, NY 14203, United States

**Keywords:** medial cuneiform, fracture-dislocation, Lisfranc injury, tibialis anterior tendon, open midfoot injury

## Abstract

Medial cuneiform fracture-dislocations are rare and typically occur in the setting of high-energy midfoot trauma. We report the case of a 30-year-old woman who sustained an open dorsomedial fracture-dislocation of the medial cuneiform with concomitant tibialis anterior tendon avulsion after a 6-m fall. She underwent emergent irrigation and debridement, open reduction, and internal fixation of the midfoot, and primary repair of the tibialis anterior tendon. Intraoperatively, a longitudinal split tear with avulsion from the medial cuneiform insertion was identified and repaired through a bone tunnel using nonabsorbable sutures. At 6-month follow-up, radiographs demonstrated maintained reduction without hardware failure or recurrent instability. The patient reported minimal pain, had no evidence of infection, and was ambulating independently. This is the first reported open dorsomedial fracture-dislocation of the medial cuneiform with acute tibialis anterior tendon avulsion.

## Introduction

Medial cuneiform injuries are uncommon and more often occur as part of a broader Lisfranc injury pattern than as isolated lesions [1, 2]. Their rarity reflects the intrinsic stability of the medial column, which is provided by the wedge-shaped osseous anatomy of the cuneiforms and the strong dorsal, plantar, and interosseous ligamentous structures of the Lisfranc complex [3, 4]. Because the plantar restraints are stronger than the dorsal restraints, traumatic displacement more commonly occurs dorsally when sufficient force is applied [4]. These injuries typically result from substantial axial, rotational, or crush mechanisms and may be subtle on initial radiographs [5, 6]. Dorsal and dorsomedial dislocations of the medial cuneiform require prompt recognition and stable anatomic reduction to restore the medial longitudinal arch and minimize the risk of chronic pain, deformity, and posttraumatic arthrosis [7].

Tibialis anterior tendon injuries in the setting of acute midfoot fractures are exceptionally uncommon. Although tibialis anterior entrapment can contribute to irreducible Lisfranc injuries [8], the combination of an open dorsomedial fracture-dislocation of the medial cuneiform and acute tibialis anterior tendon avulsion has not been described in the indexed English-language literature. We present the case of a 30-year-old woman who sustained a high-energy fall resulting in this injury pattern. She was treated with emergent open reduction and internal fixation and primary tendon repair. At 6-month follow-up, she was ambulating independently with minimal pain and had maintained alignment without radiographic evidence of hardware failure or recurrent instability. This case is compared with prior reports of medial cuneiform and related midfoot fracture-dislocations summarized in Table 1.

**Table 1:** Published reports of medial cuneiform fracture-dislocations and related cuneiform dislocations.

**Reference**	**Author/year**	**Patient age/ gender**	**Injury pattern and direction**	**Mechanism of injury**	**Treatment**	**Outcome**
[1]	Guler / 2011	32, M & 21, M	Displaced isolated medial cuneiform fracture & Nondisplaced isolated medial cuneiform fracture	Motorcycle accident & Hyperplantar flexion injury while running	Open reduction/fixation w/ 2 headless compressive screws & Below-knee plaster cast	6-month return to previous activity level & 4-month full return to activity
[2]	Aitken / 2012	39, F	Dorsomedial fracture dislocation of the first ray and medial cuneiform	Tripped over pet, fell down stairs	Closed reduction with Kirschner wire failed; ORIF w/ 4 mm cannulated screws and bone graft	Complete fusion of bone graft, minimal discomfort, and no functional limitation at 12 months
[7]	Dines / 1984	37, F	Dorsomedial dislocation of the first ray at the medial cuneonavicular joint	Slammed car breaks during a head-on collision	Closed reduction and crossed Kirschner wires	Resumed all normal activities without pain/discomfort but mild swelling at 5 months
[9]	Akan / 2013	30, F	Dorsal dislocation of the intermediate cuneiform with a medial cuneiform fracture	Fell down staircase in high-heeled shoes	Failed closed reduction; open reduction with cortical lag screws	Painless foot with normal range of motion; screws removed at 12 months
[10]	Can / 2023	20, F	Plantar dislocation of the middle cuneiform bone with medial cuneiform subluxation	70 kg iron tractor piece fell on foot	Open reduction and internal fixation with 4.5 mm cannulated screws	AOFAS 88%; back to work as a farmer with no complaints at 22 months
[11]	Levine / 1998	35, M	Plantar-lateral dislocation of the medial cuneiform	Dropped 200 lb concrete box on foot	Closed reduction	Back to normal shoes without pain/difficulty at 6 months
[12]	Hubbell / 1998	21, M	Plantar fracture-dislocation of the middle cuneiform	Wooden planks dropped on foot from 4 feet	Decompression, reduction, and stabilization with K-wires	Independent gait with occasional pain/swelling at 6 months
[13]	Brown / 1975	26, M	Distal/lateral dislocation of the medial cuneiform in tarsometatarsal fracture-dislocation	Fallen from a bridge 15.24 meters onto railroad tracks	Failed closed reduction; open reduction with Kirschner wires	Demonstrated good healing at 18 months
[14]	Hidalgo-Ovejero / 2005	48, F	Medial dislocation of the first cuneiform	Fall from a folding ladder (torsion and hyperplantar-flexion)	Failed closed reduction; reduction with pointed-reduction forceps successful with Kirschner wires	Normal gait without pain at 6 months
[15]	Lynch / 1995	44, M	Plantar medial subluxation of the medial cuneiform	Leaped over a fence, and another adult landed on the foot	Reduction and small fragment cannulated screws	None reported
[16]	Schiller / 1970	22, M	Plantar lateral dislocation of the medial cuneiform	Protruding bar of hydraulic lift struck/crushed the foot	Open reduction w/ Kirschner wires and arthrodesis	Walking without a limp and good subtalar motion at 5 months
[17]	Yutani / 1996	32, F	Medial cuneiform fracture-dislocation associated with medial and intermediate cuneiform fractures	Playing baseball (sprained ankle)	Closed reduction and immobilization with Kirschner wire	Can walk with a pattern without pain at 24 weeks
Present case	Muscat & Chernov *et al.*	30, F	Open dorsomedial fracture-dislocation of the medial cuneiform with Lisfranc instability and tibialis anterior tendon avulsion	6-m fall	Emergent irrigation and debridement, open reduction and internal fixation with three 3.5-mm cortical screws, and primary tibialis anterior tendon repair	Maintained reduction, no infection or hardware failure, and independent ambulation with minimal pain at 6 months

ORIF = open reduction and internal fixation; AOFAS = American Orthopaedic Foot & Ankle Society; M = male; F = female

## Case report

**Figure 1:**
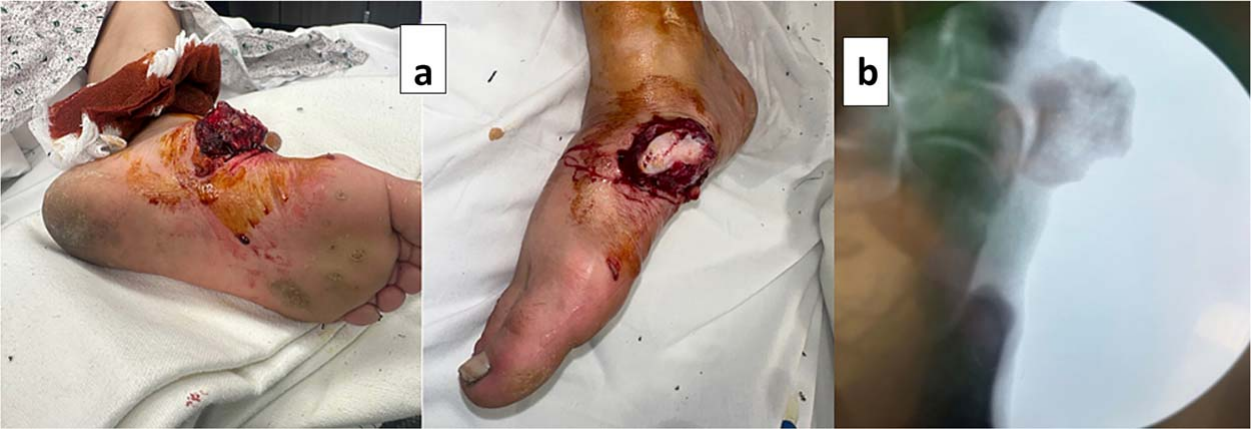
(a) Clinical photograph demonstrating an open dorsomedial wound of the right midfoot with exposed bone and articular cartilage. (b) Initial radiograph demonstrating dorsomedial displacement of the medial cuneiform relative to the navicular and base of the first metatarsal, widening of the first tarsometatarsal interval, and disruption of the medial-intermediate intercuneiform articulation.

A 30-year-old woman with a history of polysubstance use disorder, including heroin, cocaine, fentanyl, and tobacco use, presented to the emergency department after a 6-m fall. Examination revealed an open wound over the dorsomedial aspect of the right midfoot with exposed bone and articular cartilage (Fig. 1a). Dorsalis pedis and posterior tibial pulses were palpable, capillary refill was < 2 s in all digits, and sensation to light touch was intact, including in the first dorsal web space. Initial radiographs demonstrated a displaced fracture-dislocation of the medial cuneiform. The medial cuneiform was displaced dorsomedially relative to the navicular and the base of the first metatarsal, with widening of the first tarsometatarsal interval and disruption of the medial-intermediate intercuneiform articulation, consistent with severe Lisfranc instability of the medial column (Fig. 1b).

**Figure 2:**
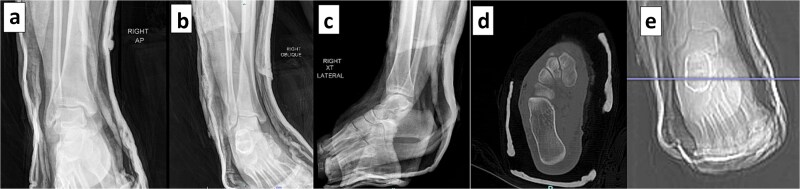
(a–c) Post-reduction radiographs obtained after bedside closed reduction and splinting demonstrate improved medial column alignment. (d–e) CT confirming adequate reduction and improved medial column alignment.

After initiation of intravenous antibiotics, closed reduction under sedation was attempted in the emergency department. Alignment improved, and the extremity was immobilized in an AO splint (Fig. 2). The patient was then taken emergently to the operating room for irrigation and debridement of the open wound and definitive fixation. The traumatic wound was extended dorsomedially, nonviable tissue was excised, and the wound was copiously irrigated. A longitudinal dorsal incision over the tarsometatarsal region was then used to expose the Lisfranc and intercuneiform joints. The second and third tarsometatarsal joints were reduced and provisionally stabilized to restore central column alignment. The first tarsometatarsal joint and the medial-intermediate intercuneiform articulation were then anatomically reduced. Two 3.5-mm cortical lag screws were placed from the medial cuneiform into the base of the second metatarsal and the intermediate cuneiform to stabilize the medial and intercuneiform joints. A third 3.5-mm cortical screw was placed across the first tarsometatarsal joint to restore congruity of the first ray.

At final wound inspection, a longitudinal split tear of the tibialis anterior tendon with avulsion from its medial cuneiform insertion was identified. The tendon was irrigated, debrided of interposed tissue, and repaired primarily using nonabsorbable sutures passed through a bone tunnel in the medial cuneiform. The capsule and skin were closed in layers. Postoperatively, the patient remained non-weight-bearing on the right lower extremity and received 48 hours of intravenous antibiotics because of the open injury.

**Figure 3:**
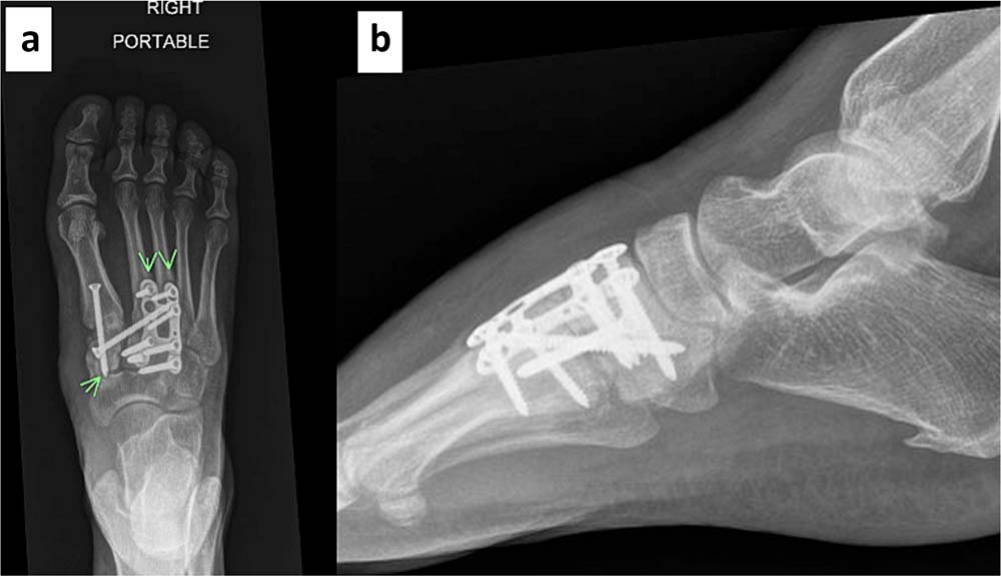
(a–b) Radiographs at 6 months demonstrate maintained reduction of the first through third tarsometatarsal joints without hardware failure or recurrent displacement.

At 6-month follow-up, the patient reported near-normal function with only minimal pain during strenuous activity. Examination demonstrated a well-aligned foot, an intact neurovascular examination, and no signs of infection. Radiographs confirmed maintained reduction of the first through third tarsometatarsal joints without hardware failure or recurrent displacement (Fig. 3). She was ambulating without assistive devices. During recovery, she also enrolled in a substance use rehabilitation program.

## Discussion

Medial cuneiform fracture-dislocations are rare midfoot injuries that usually follow high-energy trauma. As summarized in Table 1, previously reported medial cuneiform injuries consist almost entirely of isolated case reports describing plantar, dorsal, or medial displacement patterns rather than open dorsomedial fracture-dislocations [2, 7, 9–17]. Aitken and Dines described dorsomedial injuries involving the first ray/medial cuneiform complex, whereas Levine, Lynch, and Schiller described plantar or plantar-lateral displacement patterns of the medial cuneiform [2, 7, 11, 15, 16]. The present case is the first reported open dorsomedial fracture-dislocation of the medial cuneiform with concomitant acute tibialis anterior tendon avulsion.

The medial cuneiform functions as a keystone of the medial column and is constrained by strong plantar and intercuneiform ligaments, wedge-shaped osseous geometry, and the tibialis anterior insertion [11]. These anatomic constraints normally resist translational displacement; accordingly, dorsomedial dislocation is biomechanically unfavourable under physiologic loading conditions [2]. Prior reports suggest that this pattern results from a combined rotational and compressive force transmitted through a plantarflexed first ray rather than simple axial loading alone [7, 13]. Once those ligamentous restraints fail, a lever-arm effect between the first and second rays may drive the medial cuneiform dorsomedially [13].

The literature summarized in Table 1 also suggests that most patients do well after early anatomic reduction and stable fixation, whether treated with Kirschner wires or screws [2, 7, 9–17]. Our case followed that same principle but was distinguished by the open wound and the associated tibialis anterior tendon avulsion identified intraoperatively. Computed tomography (CT) should be strongly considered when high-energy midfoot trauma is accompanied by subtle or equivocal radiographs, since medial cuneiform malalignment can be difficult to appreciate on plain films [6, 16]. In addition, associated soft-tissue injury may be underrecognized. Tibialis anterior entrapment has been reported in irreducible Lisfranc injuries [8], and the present case shows that frank insertional failure of the tendon can also occur. Identification and primary repair of the tendon were important to restore dorsiflexor continuity and reinforce medial column stability [18].

Finally, surgical prognosis and recovery are influenced by patient-specific risk factors. This patient’s concurrent opioid and tobacco use increased the risk of wound complications, impaired healing, and adverse postoperative outcomes [19, 20]. Despite these risks, prompt antibiotics, thorough debridement, stable anatomic fixation, and close follow-up resulted in maintained reduction, no infection, and good short-term function at 6 months.

## Conclusion

This case demonstrates successful treatment of an open dorsomedial fracture-dislocation of the medial cuneiform with acute tibialis anterior tendon avulsion. Early recognition, prompt administration of antibiotics, urgent irrigation and debridement, stable anatomic fixation, and direct assessment of the tibialis anterior tendon were central to the favorable outcome. In severe midfoot trauma, surgeons should maintain a high index of suspicion for associated tendon injury, especially when the medial cuneiform is displaced dorsomedially or the injury is open.

## Data Availability

Data sharing is not applicable to this article because no datasets were generated or analyzed beyond those described in this case report.
